# Reduced Gray to White Matter Tissue Intensity Contrast in Schizophrenia

**DOI:** 10.1371/journal.pone.0037016

**Published:** 2012-05-15

**Authors:** Li Kong, Christina Herold, Bram Stieltjes, Marco Essig, Ulrich Seidl, Robert Christian Wolf, Torsten Wüstenberg, Marc Montgomery Lässer, Lena Anna Schmid, Knut Schnell, Dusan Hirjak, Philipp Arthur Thomann

**Affiliations:** 1 Structural Neuroimaging Group, Department of General Psychiatry, University of Heidelberg, Heidelberg, Germany; 2 Section of Geriatric Psychiatry, University of Heidelberg, Heidelberg, Germany; 3 German Cancer Research Center, Heidelberg, Germany; 4 Center of Psychosocial Medicine, Department of General Psychiatry, University of Heidelberg, Heidelberg, Germany; 5 Center for Mental Health, Klinikum Stuttgart, Stuttgart, Germany; 6 Department of Psychiatry, Charité-Universitätsmedizin Berlin, Campus Mitte, Berlin, Germany; King's College London, United Kingdom

## Abstract

**Background:**

While numerous structural magnetic resonance imaging (MRI) studies revealed changes of brain volume or density, cortical thickness and fibre integrity in schizophrenia, the effect of tissue alterations on the contrast properties of neural structures has so far remained mostly unexplored.

**Methods:**

Whole brain high-resolution MRI at 3 Tesla was used to investigate tissue contrast and cortical thickness in patients with schizophrenia and healthy controls.

**Results:**

Patients showed significantly decreased gray to white matter contrast in large portions throughout the cortical mantle with preponderance in inferior, middle, superior and medial temporal areas as well as in lateral and medial frontal regions. The extent of these intensity contrast changes exceeded the extent of cortical thinning. Further, contrast changes remained significant after controlling for cortical thickness measurements.

**Conclusions:**

Our findings clearly emphasize the presence of schizophrenia related brain tissue changes that alter the imaging properties of brain structures. Intensity contrast measurements might not only serve as a highly sensitive metric but also as a potential indicator of a distinct pathological process that might be independent from volume or thickness alterations.

## Introduction

Brain morphological changes in schizophrenia have been reported by a vast number of structural magnetic resonance imaging (MRI) studies and include ventricular enlargement [1], reductions in global and regional gray and white matter volumes [1], [2], thinning of the cerebral cortex [3]–[6], as well as altered white matter fiber tracts [7]. Compared to this continuously increasing body of research, studies evaluating differences of tissue signal properties (e.g. relaxation times or signal intensities) between patients with schizophrenia and controls remain highly underrepresented. An early region of interest (ROI) study examined T1 and T2 relaxation times in schizophrenia and found a prolongation of T2 relaxation time and to a lesser extent of T1 relaxation time pronounced in the right temporal neocortex and in the right amygdala [8]. Another ROI study investigated signal intensity within the corpus callosum in first-episode schizophrenia, non-schizophrenic psychotic disorder, and healthy controls [9]. The authors revealed that patients with schizophrenia were characterized by reduced signal intensity in several callosal subregions both when compared with healthy controls and with non-schizophrenic psychotic individuals. These findings demonstrate the presence of fundamental brain tissue changes in schizophrenia that alter the imaging properties of brain structures. However, due to methodological constraints, the aforementioned studies were restricted to predefined brain regions and thus could not account for potential alterations of signal intensity parameters in other cerebral areas.

Recently, whole brain analyses of changes in signal intensity and the effects of tissue changes on contrast properties (i.e. the gray matter/white matter intensity ratio, GWR) of neural structures have been performed in healthy aging and in Alzheimer's disease (AD) [10], [11]. The authors found increasing age to be associated with decreased contrast (i.e. GWR tending towards 1) throughout a large portion of the cortical mantle, including superior and inferior frontal, lateral parietal, superior temporal and precuneus regions, amongst others [10]. AD patients were characterized by a reduced contrast in regions known to be affected in AD comprising temporal, frontal and parietal brain regions [11]. Noteworthy, the described contrast alterations remained significant after controlling for thickness and thus indicate that – in aging and AD – GWR changes might represent a (statistically) independent measure rather than being solely attributable to cortical thinning.

In this MRI study, we investigated tissue contrast and cortical thickness in patients with schizophrenia and healthy controls accounting for potential changes throughout the entire cortical mantle. We were particularly interested in (i) whether patients with schizophrenia are characterized by an altered gray to white matter contrast, (ii) if so, whether the respective changes follow the same regional pattern as revealed for cortical thickness, and (iii) whether GWR differences between patients and controls remain after covarying for cortical thickness thus suggesting brain tissue contrast changes as a relevant biomarker of schizophrenia.

## Methods

### Participants

Eighteen patients with schizophrenia and 18 healthy controls were included in the present study. Subjects were carefully matched for age, gender, education and handedness. Clinical evaluation included ascertainment of personal and family history and detailed physical and neurological examination. None of the participants had a lifetime history of neurological or severe systemic illness, head injury or substance abuse. Handedness was assessed by means of the Edinburgh Inventory [12]. The investigations were approved by the local ethics committee and written informed consent was obtained from all participants after the procedures of the study had been fully explained. In all patients, capacity to consent was verified by an experienced psychiatrist who was not directly involved in the study. Patients were consecutively admitted to the inpatient unit of the Department of Psychiatry, University of Heidelberg, and diagnosed as suffering from schizophrenia using the German version of the Structured Clinical Interview for DSM-IV [13]. The Brief Psychiatric Rating Scale [14], the Scale for the Assessment of Positive Symptoms [15] and the Scale for the Assessment of Negative Symptoms [16] were used to assess the severity of clinical symptoms.

### MR acquisition

MRI-data were obtained at the German Cancer Research Center with a 3 Tesla Magnetom TIM Trio MR scanner (Siemens Medical Solutions, Erlangen, Germany) using a T1-weighted 3D magnetization prepared rapid gradient echo sequence (MP-RAGE, 160 sagittal slices, image matrix = 256×256, voxel size = 1×1×1 mm^3^, TR = 2300 msec, TE = 2.98 msec, TI = 900 msec, flip angle = 9°).

### Image processing

Cortical reconstruction was performed with the Freesurfer image analysis suite (http://surfer.nmr.mgh.harvard.edu/). The technical details of these procedures are described in prior publications [17]–[21]. Briefly, processing includes removal of non-brain tissue using a hybrid watershed/surface deformation procedure [21], automated Talairach transformation, intensity normalization in order to correct for MR intensity nonuniformity mainly arising from variations in the sensitivity of the reception coil and from gradient-driven eddy currents [22], tessellation of the gray matter white matter boundary, automated topology correction [23], [24], and surface deformation following intensity gradients to optimally place the gray/white and gray/cerebrospinal fluid borders at the location where the greatest shift in intensity defines the transition to the other tissue class [17], [20]. Once the cortical models are complete, a number of deformable procedures can be performed for in further data processing and analysis including surface inflation [18], registration to a spherical atlas which utilized individual cortical folding patterns to match cortical geometry across subjects [19], parcellation of the cerebral cortex into units based on gyral and sulcal structure, and creation of a variety of surface based data including maps of curvature and sulcal depth. This method uses both intensity and continuity information from the entire three dimensional MR volume in segmentation and deformation procedures to produce representations of cortical thickness, calculated as the closest distance from the gray/white boundary to the gray/cerebrospinal fluid boundary at each vertex on the tessellated surface [20]. The maps are created using spatial intensity gradients across tissue classes and are therefore not simply reliant on absolute signal intensity. The maps produced are not restricted to the voxel resolution of the original data and thus are capable of detecting submillimeter differences between groups. Cortical thickness measurements as implemented in FreeSurfer have been validated against manual measurements [3], [25] and histological analysis [26]. Further, FreeSurfer morphometric procedures have been demonstrated to show sufficient test-retest reliability across scanner manufacturers, field strengths, and other imaging parameters [27]–[29].

Signal properties were examined following the methodology described by Salat et al. [10], [11]. Gray matter intensities were measured at a depth of 35% through the thickness of the cortical ribbon from the gray matter/white matter border along the normal direction towards the gray matter/cerebrospinal fluid border. White matter intensities were extracted at 1 mm subjacent to the gray matter/white matter border along surface normal towards white matter. The aforementioned 35% sampling procedure was used to be conservatively close to the gray matter/white matter border and white matter sampling voxel (in order to allow for minimization of potential spurious effects that could arise in sampling from more remote locations and from sampling in regions of low cortical thickness). After estimation of the gray matter/white matter contrast, values were smoothed with a 30 mm Gaussian kernel using surface-based smoothing procedure that averages data across neighboring cortical locations.

### Statistical analyses

Regional effects of schizophrenia on signal intensities, tissue contrast and thickness were tested by General Linear Models (GLMs) at each vertex. To estimate the association between effects of diagnosis on the different metrics, vertex-wise values were included as per-vertex-regressors in the GLMs. This enables to test the effects of the global regressors while controlling for a vertex-wise measure. In all analyses, potential confounders including age, education, antipsychotic dosage, duration of illness and severity of psychopathological symptoms were treated as nuisance variables. Statistical maps were thresholded using the false discovery rate (FDR, p<0.05) [30]. SPSS for Windows (version 18) was used to for statistical analysis of demographical and clinical data; p values less than 0.05 were considered significant.

## Results

Demographics of all participants and clinical characteristics of the patients are summarized in Table 1. There were no significant differences in age, gender, education and handedness between groups. At the time of scanning, all patients were treated with a second generation antipsychotic according to their psychiatrists' choice (amisulpride n = 1, aripiprazole n = 1, clozapine n = 6, olanzapine n = 6, paliperidone n = 1, quetiapine n = 3). The mean chlorpromazine equivalence dosage was 660.9±479.1 mg [31]. Mean duration of illness was 25.2±9.8 years.

**Table 1:** Demographic and clinical characteristics for the patient and control groups.

	Patients (N = 18)	Controls (N = 18)	p
Demographic measures	Mean (SD)	Mean (SD)	
Age (years)	48.1 (11.3)	49.8 (7.0)	ns
Sex (M/F)	13/5	13/5	ns
Education (years)	12.3 (2.6)	13.2 (1.7)	ns
Handedness (R/L)	17/1	17/1	ns

Clinical measures			
SAPS^a^	22.2/19.1		
SANS^b^	32.7/23.2		
BPRS^c^	20.8/11.6		

a Scale for the Assessment of Positive Symptoms.

b Scale for the Assessment of Negative Symptoms.

c Brief Psychiatric Rating Scale.

### Gray matter and white matter intensity

**Figure 1:**
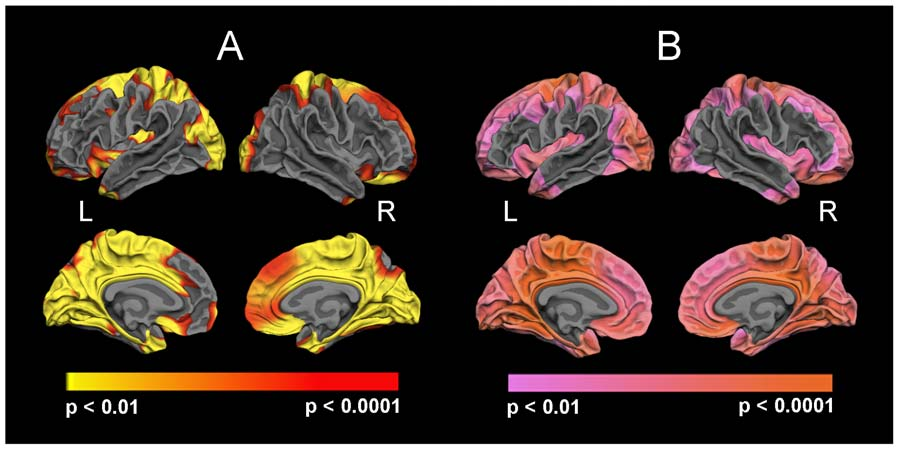
Reduced gray (A) and white (B) matter intensity in patients with schizophrenia.

Both gray and white matter intensities were significantly decreased in patients with schizophrenia throughout a large portion of the cortical mantle including frontal, temporo-parietal and occipital areas (Fig. 1A and B). Reductions in white matter intensities exceeded those in gray matter both with respect to significance and spatial extent.

### Gray to white matter intensity ratio (GWR)

**Figure 2:**
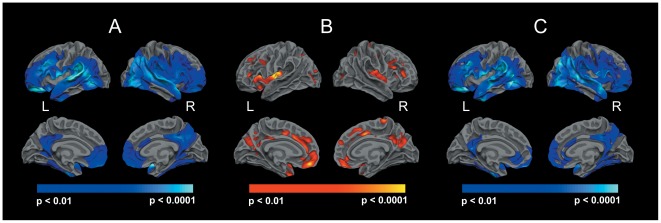
Reduced gray to white matter contrast (A) and cortical thinning (B) in patients with schizophrenia. Group differences in gray to white matter contrast remained after controlling for thickness at each vertex along the cortical mantle (C).

Gray to white matter tissue intensity contrast was bihemispherically decreased (equivalent to an increased GWR) throughout a large portion of the cortical mantle including inferior, middle, superior and medial temporal regions as well as lateral and medial frontal areas. Tissue intensity contrast was further reduced in postcentral and cingulate regions (Fig. 2A).

### Cortical thickness

In patients, cortical thickness was most significantly decreased in medial frontal regions and in the insula. In addition, significant cortical thinning was found effects were observed within the precuneus and the medial temporal lobe. (Fig. 2B). The extent of regions showing cortical thinning was clearly below that observed for decreased gray to white matter contrast.

### Association between gray to white matter contrast and cortical thickness

A regional overlap was found between intensity contrast changes and cortical thinning within the cingulate cortex, the medial temporal lobe, medial and lateral frontal areas as well as in the right insula. Group differences in gray to white matter contrast remained after controlling for thickness at each vertex along the cortical mantle (Fig. 2C).

## Discussion

This study aimed at investigating gray to white matter tissue intensity contrast and cortical thickness in patients with schizophrenia and healthy controls. Three main findings emerged: 1^st^, patients showed significantly decreased gray to white matter contrast in large portions throughout the cortical mantle with preponderance in inferior, middle, superior and medial temporal areas as well as in lateral and medial frontal regions. 2^nd^, the extent of these intensity contrast changes clearly exceeded the extent that was observed for cortical thinning. 3^rd^, contrast changes remained significant after controlling for thickness at each cortical vertex.

In contrast to extensive studies on changes in brain volume or density [1], [2], cortical thickness [3]–[6], [32], and fibre integrity [7], the effect of tissue alterations on the contrast properties of neural structures has so far remained almost unexplored in schizophrenia.

In our study, changes in tissue contrast were found to a greater extent than were changes in cortical thickness and remained significant after controlling for thickness at each vertex along the cortical mantle. These results suggest a potential role of tissue contrast changes not only as a highly sensitive metric but also as a potential indicator of a distinct pathological process that might by independent from volume or thickness alterations. In accordance with recent studies on cortical thinning in schizophrenia 3–6,32, we found patients to be characterized by reduced cortical thickness particularly in fronto-temporal cortices as well as in the insula. In our sample of patients with schizophrenia, cortical thickness was most significantly reduced within the left insular cortex. Notably, this area did not show any change in gray to white matter tissue contrast. This discrepant finding might indeed suggest some biological independence between these two measures; on the other hand, however, uncertainty remains since we cannot completely exclude the possibility that an intense regional thinning of the cortex might have affected the computation of gray matter intensity values. The few previous investigations on tissue intensity properties in schizophrenia found a prolongation of T2 relaxation time and to a lesser extent of T1 relaxation time pronounced in the right temporal neocortex and in the right amygdala [8], and a reduced signal intensity in the corpus callosum [9], respectively. Due to methodological differences, the results of the aforementioned studies as well as of our own study are not directly comparable; they, however, clearly emphasize the presence of schizophrenia related brain tissue changes that alter the imaging properties of brain structures.

It is important to note that reductions in gray to white matter contrast are far from being exclusive to schizophrenia. As recently demonstrated, patients with AD are characterized by an increased GWR in typical AD related regions, i.e. in the medial and lateral temporal, the frontal, and the parietal lobe, respectively [11]. Unlike our result of contrast changes exceeding cortical thickness reductions in schizophrenia, the mentioned study revealed losses in gray to white matter contrast and cortical thinning to be almost evenly distributed in AD. From a methodological viewpoint, this discrepancy might be at least partly explained by the MRI data acquisition at different field strengths, i.e. 3 Tesla in our study compared to 1.5 Tesla in the study of Salat and colleagues [11]. Correspondingly, a previous study reported MRI at 3 Tesla as being more sensitive to signal intensity changes than MRI at 1.5 Tesla [33]. Another explanation might be the influence of aging, since increasing age itself correlates with tissue contrast reductions to a higher degree than does advancing age with cortical thickness alterations [10]. A reduced gray to white matter contrast with aging has also been reported in prior imaging studies using different analysis techniques [34]–[36]. The strong association between increasing age and gray to white matter contrast reductions suggests that studying altered contrast properties in mental illnesses might be particularly promising in earlier adulthood. Of course, profound knowledge of the distribution and chronological sequence of brain morphological changes that occur in normal aging is crucial for understanding the mechanisms underlying these alterations and for distinguishing them from pathological processes. In this regard, great to white matter tissue contrast measurements might also be influenced by tissue specific aging, since previous structural MRI studies congruently found that the amount of gray matter starts decreasing in the early adulthood with almost linear volume reductions hereafter [37]–[41], whereas age-related white matter volume changes are characterized by a nonlinear trend with an increase continuing into the fifth decade of life followed by an accelerating white matter shrinkage with advancing age [38], [40]–[44]. With respect to the brain's maturation, previous neuroimaging studies suggest that almost every tissue compartment is changing during adolescence. These studies reported regionally distinct alterations of cortical gray matter [40], [45]–[47], white matter volume and density [42], [48], white matter microstructure [49], as well as of gyrification patterns [50]. In contrast, brain maturation effects on tissue contrast properties are so far unidentified. However, the question if and how the aforementioned brain developmental alterations might be associated with tissue contrast changes remains unresolved and should be addressed in future studies in order to enhance our understanding on how these factors relate to each other and to allow for a better characterization of disease related processes.

The precise nature of biological changes that could lead to brain tissue intensity or contrast changes remains elusive. In the present study, changes in white matter signal intensity exceeded those observed in gray matter, suggesting alterations in myelinated brain regions to mainly cause GWR changes. As reported by Salat and colleagues [10], [11], GWR changes in healthy aging and AD were also found to mainly be driven by a signal decrease in white matter tissue. Several potential mechanisms for this effect have been suggested, including de-myelinisation, changes of water content in myelin, increased ‘MR-visible’ axonal cytosol resulting from alterations in the micro-tubular density of axons, and neuronal degeneration, respectively [11], [51], [52]. Due to a loss of macro-molecular peptides or an accumulation of water, these mechanisms would lead to a decelerated spin-lattice relaxation and thus to an increase in T1 relaxation time. If GWR alterations are indeed primarily caused by white matter effects, also the differences in GWR and cortical thickness analysis become plausible, because the respective changes might differentially affect alterations in tissue contrast properties and in cortical thinning. However, although the micro-structural processes for contrast reduction remains unclear, differences in this parameter are caused by differences in spin-lattice energy transition. These differences on the other hand are most likely associated with differences in the properties of brain tissue – the lattice absorbing the energy of the excited spin ensemble. To what extent these changes are of disease-specific, pathophysiological nature is of great interest for future investigations.

There are several limitations to the current study. Besides a modest sample size and a cross-sectional design, all patients were in a more or less chronic state of their disease and thus it is not possible to draw conclusions on whether the observed changes are due to the ongoing course of schizophrenia or are already present at the time of manifestation of the disorder. However, we aimed to minimize the potential influence of chronicity in our study by covarying for the duration of illness. Further, all patients were treated with antipsychotic medication, and with respect to medication dosage, no effects on tissue intensity changes emerged in our sample. Since we did not have sufficient information on the duration of medication, we were unable to exclude this variable as a potential confound. However, previous studies examining the effect of neuroleptic treatment on brain structure have identified subcortical gray matter structures, mainly with regard to typical antipsychotics, to be particularly subjected to morphological changes [53] with the neocortex being rather spared [3], [5]. Since we analyzed changes along the cortical mantle and since patients received second generation antipsychotics, the potential influence of medication on our findings might be rather marginal. Finally, it is important to consider any potential technical confounders during the process of surface reconstruction. With respect to the results reported in the present study, one might wonder whether alterations in contrast or intensity properties might have influenced cortical thickness measures and vice versa. We aimed at minimizing the potential impact of cortical thinning on contrast measuremets by regressing out thickness at each corresponding vertex. However, we cannot fully rule out the possibility that our results might have been partly influenced by FreeSurfer inherent processing steps or technical effects such as shimming and coil sensitivity profiles.

In summary, the present findings clearly emphasize the presence of schizophrenia related brain tissue changes that alter the imaging properties of brain structures. Intensity contrast measurements might not only serve as a highly sensitive metric but also as a potential indicator of a distinct pathological process that might by independent from volume or thickness alterations.
